# Let Me See: Correlation between 5-ALA Fluorescence and Molecular Pathways in Glioblastoma: A Single Center Experience

**DOI:** 10.3390/brainsci11060795

**Published:** 2021-06-16

**Authors:** Francesco Maria Calamo Specchia, Matteo Monticelli, Pietro Zeppa, Andrea Bianconi, Francesco Zenga, Roberto Altieri, Beatrice Pugliese, Giuseppe Di Perna, Fabio Cofano, Fulvio Tartara, Luca Bertero, Paola Cassoni, Antonio Melcarne, Michele Maria Lanotte, Diego Garbossa

**Affiliations:** 1 UOC Neurosurgery, Head and Neck Department, Azienda Ospedaliero-Universitaria di Parma, 43126 Parma, Italy; fra.calamo@gmail.com; 2 Neurosurgery Unit, AOC Città della Salute e della Scienza, Department of Neuroscience “Rita Levi Montalcini”, University of Turin, 10124 Turin, Italy; mmonticelli89@gmail.com (M.M.); pietro_zeppa@yahoo.it (P.Z.); andrea.bianconi@edu.unito.it (A.B.); zengafra@hotmail.com (F.Z.); beatricepugliese2202@gmail.com (B.P.); fabio.cofano@gmail.com (F.C.); amelcarne@gmail.com (A.M.); michele.lanotte@unito.it (M.M.L.); dgarbossa@gmail.com (D.G.); 3 Department of Neurological Surgery, Policlinico “G. Rodolico”, University Hospital, 95124 Catania, Italy; reberto.altieri.87@gmail.com; 4 Neurosurgery Unit, Istituto Clinico Città Studi, 20131 Milan, Italy; tartarafulvio@gmail.com; 5 Pathology Unit, AOC Città della Salute e della Scienza, 10126 Turin, Italy; luca.bertero@unito.it (L.B.); paola.cassoni@unito.it (P.C.)

**Keywords:** glioblastoma, 5-ALA, fluorescence, molecular pathways, extent of resection, IDH1, MGMT

## Abstract

Background: Despite the aggressiveness of multimodal treatment, glioblastoma (GBM) is still a challenge for neurosurgeons, neurooncologists, and radiotherapists. A surgical approach is still a cornerstone in GBM therapeutic management, as the extent of resection is strongly related both to overall survival and progression-free survival. From this perspective, the use of photodynamic molecules could represent an interesting tool to achieve maximal and safe resection. Being able to trace the lesion’s edges, indeed, could allow to improve the extent of resection and to minimize residual tumor while sparing normal tissue. The use of 5-aminolevulinic acid (5-ALA) as a photodynamic tracer is well established due to its strict correlation both with cellularity and metabolic activity of the GBM cell clones. Objective: Our study aims to define whether a different molecular asset of GBM (especially investigating *IDH 1/2* mutation, proliferation index, and MGMT promoter methylation) results in different fluorescence expression, possibly because of differences in metabolic pathways due to different genotypes. Methods: Patients undergoing surgery for GBM removal at our Institute (Dep. Of Neurosurgery, Ospedale Città della Salute e della Scienza, University of Turin, Italy) were retrospectively reviewed. Patients with histological diagnosis confirmation and to whom 5-ALA was given before surgery were included. The whole surgical procedure was recorded and then analyzed by three different people (a medical student, a resident, and a senior surgeon with an interest in neurooncology and experience in using 5-ALA) and a score was assigned to the different degrees of intraoperative fluorescence. The degree of fluorescence was then matched with the genotype. Results: A trend of grade 2 fluorescence (i.e., ”strong”) was observed in the *IDH 1/2* wild-type (WT) genotype, suggesting a more intense metabolic activity in this particular subgroup, while, no or weak fluorescence was observed more often in the *IDH 1/2* mutated tumors, suggesting a lower metabolic activity. No relations were found between fluorescence grade and MGMT promoter methylation or, interestingly, cellularity. As a secondary analysis, more epileptogenicity of the *IDH 1/2* mutated GBM was noticed, similarly to other recent literature. Conclusion: Our results do not support the use of 5-ALA as a diagnostic tool, or a way to substitute the molecular profiling, but confirm 5-ALA as a powerful metabolic tracer, able to easily detect the pathological cells, especially in the IDH WT genotype, and in this perspective, further studies will be necessary to better describe the metabolic activity of GBM cells.

## 1. Introduction

The prognosis of patients suffering from glioblastoma (GBM) still remains poor despite presently employed multimodality, incorporating surgical resection followed by radiotherapy and chemotherapy [1,2,3,4].

Surgery represents a cornerstone in the therapy of this tumor since prognosis strictly correlates with the extent of resection (EoR) [4,5,6,7].

During the last few decades, molecular markers have been discovered and established to improve prognostic stratification of these malignancies, aiming to identify patient-tailored adjuvant post-surgery therapeutic regimens [8,9,10].

Simultaneously, the use of fluorescence in glioma surgery improved radiological and clinical results related to the extent of resection because of its capability to involve not only the enhancing nodule but also the infiltrating tumor outside the MR enhancing lesion [11,12,13,14].

Our study aimed to define whether a different molecular subset of GBM (especially in terms of *IDH 1/2* mutation status, proliferation index, and *MGMT* promoter methylation) could result in a different fluorescence expression, possibly because of differences in metabolic pathways which could reflect a different genotype.

## 2. Materials and Methods

### 2.1. Population

We prospectively collected a series of consecutive patients (age >18 years) with suspected GBM on pre-op MRI, who underwent surgical resection at our institution at the Department of Neurosurgery—Città della Salute e della Scienza, University of Turin after evaluation of inclusion/exclusion criteria.

Inclusion criteria were: (1) histological confirmation of newly diagnosed GBM (grade IV sec. WHO 2016); (2) the availability of *IDH 1/2* mutation evaluation; (3) the availability of MGMT promoter methylation evaluation.

Exclusion criteria were: (1) patients harboring WHO grade II and/or grade 3 diffuse astrocytic tumor or other astrocytic tumors; (2) no complete availability of clinical, radiological, intra-operative, and histological data.

### 2.2. Procedure

**Figure 1:**
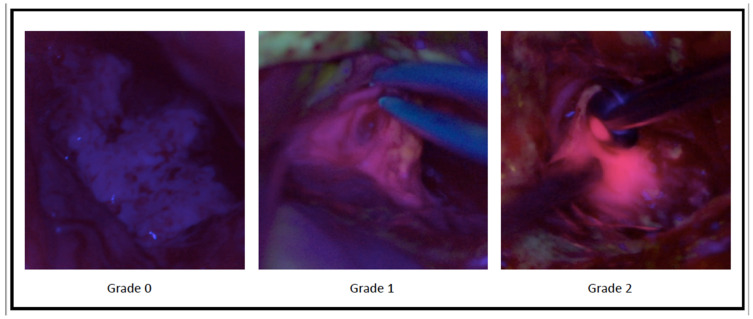
Classification of 5-ALA fluorescence grading scale according to Stummer et al. [16].

Surgical procedures were performed by 3 senior surgeons with an interest in neurooncology (F.Z., A.M., D.G.). After achievement of informed consent, we administered 5-aminolevulinic acid (5-ALA) 3 h before the procedure (20 mg/kg). All the procedures were performed by using a Pentero Zeiss Microscope with a proper UV 400 nm filter for the visualization of 5-ALA fluorescence in a dark operative room (OR) to avoid potential biases due to external light. Every single procedure was recorded and then reviewed by 3 different people (a senior neurosurgeon, a neurosurgery resident, and a medical student), giving a score to the different fluorescence grades. A scale previously described by Stummer et al. (Figure 1) was used: Grade 0—no fluorescence, Grade 1—weak fluorescence, Grade 2—strong fluorescence [15]. If the three reviewers disagreed, the fluorescence grade that had obtained at least two out of three preferences was used. Cases with complete interobserver disagreements were found to be misleading and eliminated. Inter-observer concordance was considered in order to quantify the eventual bias related to different surgeons who estimated the fluorescence grade.

### 2.3. Post-Operative Management

All patients underwent postoperative MRI with gadolinium 48 h after surgery in order to evaluate the residual tumor volume. The histopathological diagnosis and molecular data were provided by our dedicated neuropathologist, focusing on *MGMT* promoter methylation status, *IDH 1/2* gene status (always confirmed by sequencing), mitotic count, and Ki67 proliferation index. As per department protocol, after histological confirmation of GBM, patients were sent to multidisciplinary evaluation for determining adjuvant therapies.

### 2.4. Statistical Analysis

Categorical variables were presented as absolute numbers and percentages, while quantitative variables were presented as mean values (standard deviation). Inter-observer concordance was evaluated with Fleiss’ Kappa. Correlation between groups for the categorical variables was obtained using the chi-square test or the exact Fisher test if at least one cell had an expected value <5. For quantitative variables, the Shapiro-Wilk normality test was used and the test between groups was performed using variance analysis and t-test if the variables were normally distributed, otherwise we used the respective non-parametric tests of Kruskal–Wallis and Mann–Whitney. In the case of multiple comparisons, the Bonferroni correction was used.

All tests were performed at two-tail, with a significance level (α) at 5%. All the analyses were made using the STATA 14.1 SE software (STATACorp, College Station, TX, USA).

## 3. Results

A total number of 44 (28 M, 16 F) met the inclusion criteria and were considered for statistical analysis. The mean age was 65.7 y (ranging from 37 to 81 years). The presence of a progressive neurological deficit was the most frequent presenting symptom observed in our sample (54%), followed by seizures (12%), pure motor deficit (2%), and headache (2%). In 23% of the patients, mixed symptoms (headache/progressive neurological deficit, progressive neurological deficit/seizures, etc.) resulted to be the presenting symptom (Table 1).

### 3.1. Interobserver Concordance

In line with the simplicity of the adopted fluorescence grading system, the inter-observer concordance was almost perfect (Fleiss’ Kappa 0.92). A complete disagreement between the observers was never registered.

### 3.2. IDH-1/2 Mutation Status

**Figure 2:**
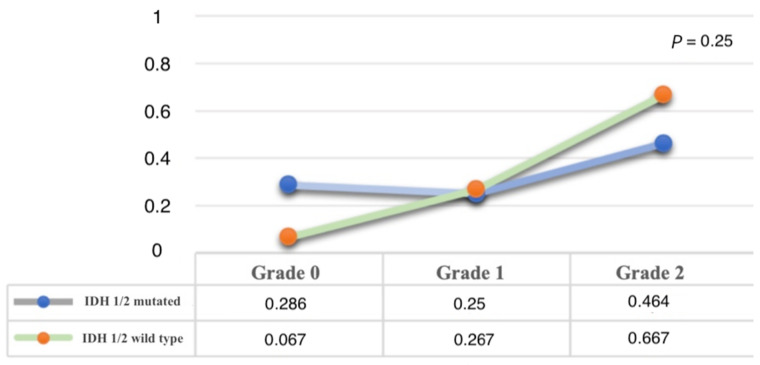
Correlation between IDH-1 status and fluorescence grade.

The results are summarized in Table 1. Overall, we observed no statistical correlation between fluorescence grade and *IDH-1/2* genotype (mutation/wild type) (*p* = 0.25). Despite this, we noticed that 10 IDH-wt patients (66%) out of 15 showed a Grade 2 fluorescence (intense nodular), while a Grade 0 fluorescence was more often observed in the *IDH 1/2*-mutated group (88.89% vs. 11.11%). A similar value was observed for the Grade 1 (faint fluorescence) group (Figure 2).

Despite more variables were used for stratification, no statistical correlation between mitotic index, number of mitoses, and *IDH 1/2* status were found (*p* = 0.13 and 0.96, respectively) (Table 1).

Further analyses investigating associations between *IDH-1/2* status and clinical presentation were conducted. Specifically, although neurological deficits, motor deficits, headache, and seizures did not show statistically significant associations, a huge difference was observed in the *IDH-1/2* mutation group, being higher than the number of patients with seizures as clinical onset (25% vs. 0% *p =* 0.08).

### 3.3. Role of Mitotic Index, Gender, Age

As reported in Table 2, differences in fluorescence grade were not related to mitotic index (*p* = 0.93) and no significant differences were found stratifying fluorescence according to gender (*p* = 0.708) or median age (66.6 ± 14.3 vs. 65.83 ± 11.3 vs. 65 ± 12.7, respectively, in Grade 0, 1 and 2).

### 3.4. MGMT Promoter Methylation

Focusing on MGMT mutations, by matching fluorescence grade with the methylation state of *MGMT* promoter, a trend for a higher grade of fluorescence (Grade 2) among un-methylated GBMs (60% un-methylated vs. 40% methylated, *p* = 0.11) was found.

## 4. Discussion

The aim of the study was to assess whether different fluorescence grades expressed by GBM cells could be related to a particular histopathological/molecular subtype (methylation of *MGMT* promoter, mitotic count, proliferation index, and *IDH-1/2* gene status), thus, helping to predict the more probable genotype according to detected intra-operative fluorescence.

Nowadays, 5-ALA guided tumor resection is well established in literature, especially for GBM, even though further experience—all over the neurooncology field—has been reported (i.e., low-grade gliomas LGG, meningiomas, metastasis, lymphoma biopsies) [17,18,19,20,21,22,23].

For a better interpretation of fluorescence patterns, many authors focused their attention on the metabolic activity of the tumor [18,24].

The most studied relationship between fluorescence grade and biological behavior is the correlation between fluorescence and cellular density, considering the difference in cellularity between the enhancing nodule and tumor border/infiltrated parenchyma. All the observations in the literature found stronger fluorescence to be directly related to cellularity but also to a different genotype between cells of the same tumor (more “staminal” biomarkers of the core-cells compared to more differentiated cells in the tumor border) [24,25]. This corresponds to stronger fluorescence in the tumor “core” and weaker fluorescence in the margins [26].

While there is still no in vitro demonstration, a relationship between 5-ALA fluorescence and IDH mutation is theoretically possible, as IDH is an enzyme of Krebs cycle, catalyzing the formation of α-ketoglutarate from the de-carboxylation of D-isocitrate. Once formed, α-ketoglutarate enters the Krebs cycle, forming succinyl-CoA. This metabolite, together with glycine, is involved in the ALA metabolic pathway as a precursor in the porphyrin cycle [17].

Hence, IDH-mutated tumors could show different activity in porphyrin pathways and different sensibility to external precursors as 5-ALA, thus, consequently, different types of fluorescence under UV light after 5-ALA administration.

Few data regarding the relationship between *MGMT* promoter methylation and porphyrin pathways are available in the literature.

Jaber et al. did not find any correlation between MGMT methylation status and any grade of fluorescence expression in LGG, while, after reviewing the literature concerning high-grade gliomas, only a case report about a recurrent epithelioid GBM was found. The authors described the progressive shift of the tumor (at his 3rd recurrence) from epithelioid to classic GBM pattern, with loss of MGMT methylation and related intense fluorescence expression after 5-ALA administration [17].

Focusing only on primary GBMs, hence excluding possible biases due to oligodendroglial tumors or low-grade components, the presented series does not show associations between fluorescence and *IDH-1/2* mutations, resulting in 66.7% of patients with Grade 2 fluorescence in the IDH-wt group vs. 46.4% of Grade 2 in the *IDH-1/2* mutated group (*p =* 0.25). As a matter of fact, GBM usually exhibits fluorescence, despite the specific molecular status.

However, although no statistical significance emerged, it could be interesting to observe a trend towards a higher expression of Grade 2 fluorescence in the IDH-wt group when compared to Grades 0/1 (66.7% vs. 6.7% and 26.7%, respectively), as observed in the literature reports [17]. Conversely, Grade 0 fluorescence was more often observed in the *IDH-1/2* mutated group (28.6% vs. 6.7%). Probably, a different genetic/metabolic substrate involving *IDH-1/2* could be argued to explain the data being poor uptake and metabolization of 5-ALA as the reason for poor fluorescence in the *IDH-1/2* mutated group compared to wild-type tumors. The difference in fluorescence, in this case, did not seem to be due to differences in tumor cellularity/proliferation. Indeed, as we have seen, the secondary analysis showed that there were no differences between the three grades of fluorescence in mitotic count (34.8 ± 12.1 vs. 37.2 ± 19.3 vs. 35 ± 15.6).

This means that, in a pool of primary GBMs, even in presence of high cellularity, false negativity to 5-ALA (Grade 0 fluorescence) could be observed and more often in the IDH-mutated genotype, pointing to a different metabolic asset in this subpopulation of GBMs. As recently showed by Rampazzo et al., 5-ALA positive cell population is highly variable even within the same tumor [26]. Comparing 5-ALA positivity to histological and cytological observations, they found a non-perfect concordance, demonstrating that a pool of GBM cells could avoid 5-ALA uptake. This mechanism is still unclear and the proposed hypotheses were a poor vascularization or presence of a 5-ALA-uncaptant cell clone [15].

As predicted, no correlation was found between *MGMT* promoter methylation and fluorescence, with 60% of patients with Grade 2 fluorescence in the un-methylated group vs. 40% of patients with Grade 2 fluorescence in the methylated group. Thus, the observation in the previously reported case report of simultaneous appearance of fluorescence and un-methylation of MGMT promoter should be probably explained as a more complex genetic shift towards increased cellular metabolism, more characteristic of classic GBM type, compared to the epithelioid variant [27].

In our series, even if not statistically significant, a higher number of patients presenting with seizures were reported in the *IDH-1/2* mutated group (25% of seizures as first symptoms in the *IDH-1/2* mutated group vs. 0% in the *IDH-1/2* wild-type, with a *p* < 0.08).

This topic has been previously investigated and reported in literature. In a recent case series, Yang et al. [3] examined gliomas associated with seizure (GAS), Grades II–IV, and showed a potential association with molecular profiles. In that series, a significant correlation has been found between IDH mutation and seizures as first symptoms. This could be due to the higher D-2-hydroxyglutarate concentration), which is structurally similar to glutamate, hence, capable of activating the NMDA receptors and lowering the seizure threshold [11,16,28]. However, other factors could contribute to lowering the epileptic threshold, like tissue damage/necrosis/hemosiderine deposits [29,30].

Notably, the population with *IDH-1/2* mutations and seizures as first symptoms is quite low, probably due to the fact that the analysis was focused on GBM only; hence, the non-statistical significance could be due to the lack of a proper sample size. 

As a matter of fact, a higher percentage (80%) of patients with seizures in the IDH mutated population has been reported in the literature when compared to the wild-type population (20%) [31,32].

This result, also showed by our series, could confirm a more epileptogenicity of secondary GBM, probably due to activation of metabolic pathways closer to the low-grade glioma ones.

### Limitations

Limitations of the present study are represented essentially by the impossibility to standardize and measure the difference in fluorescence grade. To avoid this kind of bias, all the procedures were performed in a dark room using the same setting, a very simple scale of the evaluation was adopted, and different people blinded to each other assigned the score. Moreover, the high rate of inter-observers’ concordance further reduced this bias.

Another relevant limitation is related to the restricted number of patients who were included in the study. As the statistical analysis shows, indeed, although the aforementioned trend toward a relationship between a determined grade of fluorescence and IDH genotype of the tumor could be considered interesting, it did not result to be statistically significant. However, this could be due to the chi square’s sensibility to a small sample size. According to the Solvin rule, when able to determine the correct sample size, at least 149 patients should be enrolled to provide statistical power to the reported findings.

Moreover, the higher prevalence of the IDH 1 mutated group in the series—although discordant with the literature—could be due to a selection bias related to the prospective collection of data and to the respect of the inclusion/exclusion criteria. Obviously, this represents a limitation that, albeit reduces the possibility to extend conclusions to the entire population but, however, allows to pose bases for further studies.

Further studies in this direction could be performed using quantification of fluorescence, namely by detecting the concentration of protoporphyrin IX with an intra-operative optic fiber spectrometer, as already described in the literature [12,24].

Absolute concentration of protoporphyrin IX could represent, in that setting, an objective parameter to assess the score. This technique has already been proven effective to quantify 5-ALA metabolic activity even in areas with negative fluorescence under intra-operative microscope, hence, restricting the true 5-ALA negative pool and the false-negative quote [12,24].

## 5. Conclusions

The use of 5-ALA in high-grade glioma surgery is a valid tool to define extension, margins, and infiltration during the procedure, showing an excellent correlation with the histological degree expressed by the tumor, in accordance with what emerged in the literature.

Our analysis on a series of GBMs has shown an association that, although not statistically significant, suggests a stronger fluorescence in the *IDH-1/2*wt group and a poorer fluorescence in the *IDH-1/2* mutated group. No correlation was detected between the MGMT promotor methylation and the degree of fluorescence.

Furthermore, we observed an increased epileptogenicity in the IDH mutated group, supporting other literature results.

Finally, although the reported results could not allow support for the employ of 5-ALA for the diagnosis and identification of GBM molecular profiles, they confirm and support the use of 5-ALA as a “metabolic” tracer. Thus, from this perspective, these results could be used for further pre-clinical studies on the metabolic activity of GBM cell lines, to better understand genetics, and metabolism of GBMs.

## Figures and Tables

**Table 1:** Descriptive results.

	*IDH-1/2* Mutation	*IDH-1/2* Wild-Type	
	*n* = 28	*n* = 16	*p*
Male (*n* = 28)	20 (71.4%)	8 (50%)	0.24
Female (*n* = 16)	8 (28.6%)	8 (50%)	0.24
Age	66.4 ± 2.5	63.3 ± 3	0.43
**Presentation**			
Progressive neurological deficit	21 (75%)	12 (80%)	0.96
Pure motor deficit	1 (3.6%)	3 (20%)	0.11
Headache	3 (10.7%)	4 (26.7%)	0.22
Seizures	7 (25%)	0	0.08
**Methylation of *MGMT*** **Promoter**	17 (60.7%)	7 (46.7%)	0.38
Methylation degree	*n* = 14	*n* = 7	0.72
High	5 (35.7%)	2 (28.6%)	
Intermediate	4 (28.6%)	1 (14.3%)	
Low	5 (35.7%)	4 (57.1%)	
**Fluorescence Grade**			0.25
Grade 0—No fluorescence	8 (28.6%)	1 (6.7%)	
Grade 1—“Weak” fluorescence	7 (25%)	4 (26.7%)	
Grade 2—“Strong” fluorescence	13 (46.4%)	10 (66.7%)	
**Mitotic Index**	31.8 ± 11	43.9 ± 21.1	0.13
**Mitosis**	11.2 ± 7.4	11.0 ± 6.7	0.96

**Table 2:** Correlation between fluorescence grade and mitotic index.

Correlation between Fluorescence Grade and Mitotic Index	*p* = 0.93
Grade 0—No fluorescence	34.8 ± 12.1
Grade 1—“Weak” fluorescence	37.2 ± 19.3
Grade 2—“Strong” fluorescence	35.0 ± 15.6

## Data Availability

Not applicable.
